# Psychiatric presentation of Gerstmann–Sträussler–Scheinker disease with 9-OPRI mutation in *PRNP gene*: a case report

**DOI:** 10.1186/s13256-025-05700-6

**Published:** 2025-12-01

**Authors:** Ecem Su Atil, Philipp Lenz, Bernhard T. Baune, Pegah Sarkheil

**Affiliations:** 1 Department of Psychiatry, University Hospital Münster, Münster, Germany; 2 Central Palliative Medicine Unit, University Hospital Münster, Münster, Germany

**Keywords:** Prion disease, Gerstmann–Sträussler–Scheinker syndrome (GSS), Neurodegenerative diseases, Neuropalliative care, Case report

## Abstract

**Background:**

Gerstmann–Sträussler–Scheinker syndrome is an extremely rare autosomal-dominant neurodegenerative disease that presents with a broad spectrum of neurological and psychiatric symptoms. Systematic case reports, which document genetic mutations and phenotypic variations, are a significant contribution to improving the diagnosis and approach of these rare diseases.

**Case report presentation:**

A 42-year-old White woman with a confirmed diagnosis of Gerstmann–Sträussler–Scheinker syndrome was admitted to the psychiatry department due to severe confusion and episodes of agitation. The patient had been experiencing marked irritability and daytime restlessness, as well as severe insomnia that resulted in nocturnal falls. She exhibited fluctuating stereotypical behavior and disorientation, failing to recognize her husband and child.

The aim of the psychiatric intervention was to enhance the patient’s quality of life by alleviating acute psychiatric symptoms through pharmacological treatment. Following successful adjustment of medication, the patient was discharged from inpatient treatment after approximately 1 month, demonstrating marked improvement in her clinical condition. However, 3 months later, she was readmitted to the hospital with progression of symptoms, including sleep disturbance, agitation, gait instability, and severe imbalance, which led to recurrent falls. She also developed dysarthria, which substantially impaired her verbal communication. The progressive neurodegenerative course of her illness ultimately necessitated extensive support from her family at home, imposing considerable emotional, physical, and financial strain on the caregivers. A collaborative approach with the neuropalliative care team was initiated to optimize symptom management and to develop individualized models of care.

**Conclusion:**

The patient presented with atypical and intermittent speech disturbances, tremors, and bradykinesia, which were initially misdiagnosed as conversion disorder. A positive family history prompted PRNP genetic testing, leading to the definitive diagnosis of Gerstmann–Sträussler–Scheinker syndrome. The management of cases in patients with incurable prion diseases poses distinct challenges, including unpredictable clinical progression and highly individualized symptom trajectories. A multidisciplinary approach is essential to address psychiatric manifestations, navigate legal and ethical considerations, and ensure comprehensive professional care. The development of adaptable care models that respond to the complex needs of these rare conditions remains a critical priority.

## Introduction

Neurodegenerative diseases are most commonly associated with older adults. However, when they manifest in young adults, they present distinct and multifaceted challenges, particularly in the context of mental health care. While conditions such as Huntington’s disease, early-onset Parkinson’s disease, and frontotemporal dementia are comparatively well recognized, other rare disorders with early-onset neurodegeneration present substantial challenges to effective disease management.

We present a case of Gerstmann–Sträussler–Scheinker (GSS) disease, an exceptionally rare autosomal dominant neurodegenerative disorder that usually manifests in the fourth or fifth decade of life. This fatal condition results from genetic mutations leading to the accumulation of abnormal prion proteins.

As a consequence of progressive neuronal loss, these disorder manifests with a broad spectrum of neurological symptoms, including cerebellar ataxia, dysarthria, cognitive decline, motor disturbances (such as myoclonus, spastic paraparesis, and parkinsonism), and alterations in sleep. Furthermore, patients frequently exhibit psychiatric manifestations, including depression, anxiety, apathy, and irritability, with psychosis occurring in a subset of cases. Behavioral disturbances, including impulsiveness, aggression, disinhibition, and socially inappropriate conduct, may also be observed. These symptoms may arise directly from the neurobiological impact of the disease or secondarily as responses to the psychological burden of a progressive, debilitating condition. In any case, such manifestations are often heterogeneous, fluctuating, and profoundly distressing for both patients and their families.

Men and women appear to be affected equally. The phenotypic heterogeneity, combined with the limited coverage of recently established molecular genetic testing, complicate accurate estimation of disease prevalence, which is thought to range from 1 in 10 million to 1 in 100 million individuals [1]. Misdiagnosis with other neurological or psychiatric disorders remains highly likely [2–5].

Systematic case reports documenting genetic mutations and phenotypic variability provide an important contribution to advancing the diagnostic accuracy of this exceptionally rare disorder. In this report, we present the case of a patient with GSS who required hospitalization and integrated psychiatric and palliative care in the context of complex mental health and psychosocial complications.

## Case report

A 42-year-old White woman from North Rhine–Westphalia, Germany, with a confirmed diagnosis of GSS, was admitted to the Department of Psychiatry and Psychotherapy at the University Hospital Münster in August 2023 due to an episode of severe confusion and agitation that had progressively worsened over several days and could no longer be managed at home. This admission followed a 4-day stay in the Department of Neurology, where treatment focused on optimizing her medication regimen. According to her caregiver, the patient exhibited persistent and escalating nervousness and restlessness. She also experienced profound nocturnal sleep disturbances, resulting in frequent nighttime restlessness and recurrent falls. During the day, her clinical picture was characterized by mood swings and extreme emotional expressions. In the afternoons, she occasionally demonstrated transient periods of improved cognitive clarity; however, despite these brief intervals, she continued to display pronounced stereotyped behaviors and disorientation, including failure to recognize her husband and child.

### Medical history

According to medical records, the patient first presented to the Department of Neurology at the University Hospital Münster in January 2019 with intermittent tremor, dysarthria, and psychomotor slowing. At that time, diagnostic investigations (including magnetic resonance imaging [MRI], electroencephalography [EEG], and lumbar puncture) yielded unremarkable results, and a diagnosis of conversion disorder was made.

At a physical examination in March 2022, the patient demonstrated progressive deterioration of speech, characterized by impairments in word finding and sentence construction. During the same period, she experienced recurrent falls at home due to gait disturbances.

**Fig. 1:**
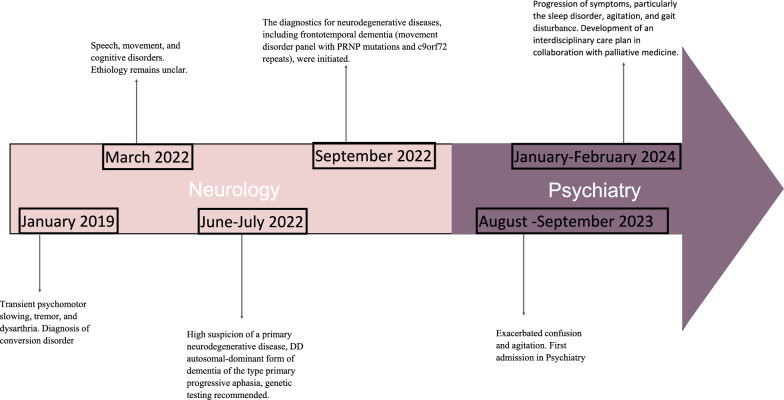
Timeline of symptoms and diagnoses

In January 2023, a diagnosis of GSS was established and confirmed through molecular genetic testing at the Department of Molecular Genetics, University Hospital Münster. Long-read sequencing demonstrated that both the patient and her father were heterozygous, carrying an insertion of nine additional octapeptide repeat units within the typical region of the PRNP gene (Fig. 1).

### Family history

The patient’s father, a British military veteran, and her paternal grandmother both developed a comparable constellation of symptoms, which culminated in premature death at relatively young ages. The patient is an only child and has no siblings.

### Physical and psychopathological examination

Neurological examination revealed dysarthria and reduced muscle strength across all extremities, with preserved deep tendon reflexes in both the upper and lower limbs. Postural and resting tremors, together with stereotyped movements, were observed in the upper extremities. The Romberg sign was positive. During the patient’s initial 2-month hospitalization, serial comprehensive psychopathological assessments were conducted, demonstrating emotional instability, sleep disturbances, restlessness, diminished expressiveness, and impairments in concentration and memory.

Table 1**:** Chronological overview of investigation findings.

**Table 1:** Chronological list of investigation findings

Date	Investigations	Results
January 2019	Cranial MRI (with contrast) Lumbar puncture EEG	Unremarkable findings
November 2021	Cranial CT	Cerebral atrophy; widened CSF spaces
January 2022	Cranial MRI	Mild, nonspecific cerebral atrophy
June 2022	Neuropsychological assessment	Severe deficits across memory, executive functions, language, and movement skills
	EEG	Mild-to-moderate encephalopathy
	ENG	Mild axonal tibial neuropathy bilaterally
January 2023	Molecular genetic testing	Gerstmann–Sträussler–Scheinker syndrome (GSS) confirmed

CSF, cerebrospinal fluid; CT, computed tomography; ENG, electronystagmography

## Treatment and follow-up

At the time of psychiatric referral, the patient exhibited such severe agitation and profound disorientation that her symptoms could no longer be managed at home. The primary objective of psychiatric intervention was to alleviate acute symptoms and enhance quality of life. To regulate the sleep–wake cycle, eszopiclone (2 mg nightly) and extended-release quetiapine (200 mg in the evening) were initiated. Daytime restlessness was managed with quetiapine (25 mg, administered four times daily).

Palliative medical care was initiated in parallel with psychiatric treatment. To mitigate agitation and reduce the risk of falls, a dementia-friendly environment was established. The patient received intensive psychiatric nursing care, incorporating structured interpersonal contact, distraction techniques, and orientation support. In addition, the family was counseled on adapting the home environment to accommodate the patient’s evolving needs.

Following these interventions and successful adjustment of her medication regimen, the patient was discharged after approximately 1 month of inpatient treatment in a markedly improved clinical condition.

The patient was readmitted approximately 3 months later. Her husband, serving as the primary caregiver, reported a progression of symptoms, most notably sleep disturbance, agitation, and gait impairment, which led to recurrent falls. At this stage, the patient was unable to walk without assistance due to severe imbalance and impaired gait. Verbal communication was restricted as a result of dysarthria. Pharmacological management was adjusted with the initiation of escitalopram (10 mg) and valproate to address anxiety and emotional instability. At home, the patient required extensive support from her family, imposing a considerable emotional, physical, and financial burden on the caregivers.

## Discussion

In this report, we present a genetically confirmed case of GSS with an inherited 9-OPRI mutation (insertions of nine octapeptide repeat units) in the PRNP gene. Various mutations of the PRNP genes have been identified in GSS cases worldwide. To our knowledge, this is Germany’s first case report of confirmed GSS with 9-OPRI mutation by PRNP analysis. Notable GSS-related PRNP mutations include P102L, P105L, A117V, Q160X, F198S, Q217R, Y218N, Y226X, and Q227X, among which P102L is the most common. P102L is considered the pathogenetic mutation of GSS [6]. To date, only four cases of 9-OPRI have been reported in the medical literature. A significant challenge with prion diseases is their frequent misdiagnosis. In this case, the diagnosis was only confirmed 6 years after the onset of symptoms and following a misdiagnosis of conversion disorder. Recent advancements in next-generation sequencing technologies offer the potential for faster and more precise genetic profiling for patients with various types of dementia. Early genetic counseling and screening could be considered early to enhance the accuracy of differential diagnoses.

In the early stages, our patient experienced major problems with unsteady gait and sleep disruption, which were indicative of cerebellar dysfunction. Manifest cognitive dysfunction and extrapyramidal symptoms gradually appeared 3 years after onset. This is not uncommon in prion diseases, with psychiatric symptoms as the initial presenting symptom in 87% of patients with prion diseases [7, 8]. Brain imaging and other diagnostic tests such as EEG and lumbal puncture do not provide clear diagnostic results. PRNP gene testing might be considered in young patients between 35 and 55 years of age with a positive family history of similar symptoms.

Early diagnosis, timely management, and comprehensive clinical care are essential for patients with GSS, as no disease-modifying therapy is currently available [9] [10, 11]. The management of psychiatric symptoms in neurodegenerative diseases is particularly challenging due to the progressive course of the disease. Pharmacological treatments are necessary to address mood and anxiety-related symptoms [12], while progressive cognitive decline often limits the utility of conventional psychotherapeutic approaches. This underscores the need for adaptive therapeutic approaches specifically designed to accommodate cognitive impairments.

Ethics consultations and interdisciplinary case conferences are integral in navigating the complex ethical and legal issues associated with progressive neurodegenerative conditions, including questions of accommodation, guardianship, and appropriate care settings. The progressive loss of cognitive capacity in prion diseases raises profound concerns regarding consent, autonomy, and the role of families in decision-making. This case highlights a critical gap in the management of prion diseases, which are marked by incurable and rapidly progressive cognitive decline often accompanied by severe disorientation and anxiety. The lack of systematic clinical trials continues to limit the development of standardized treatment approaches. The emerging field of neuropalliative care offers promising strategies by emphasizing symptom control and the development of individualized care models. However, greater attention is needed in both clinical practice and research to address the unique challenges posed by these disorders [13, 14].

## Conclusion

The patient initially presented with an atypical and intermittent speech disorder, tremors, and slowed movements, which led to a misdiagnosis of conversion disorder. A positive family history provided the basis for pursuing PRNP genetic analysis, which confirmed the final diagnosis of GSS. The care of a patient with incurable prion disease presents unique challenges. A multidisciplinary approach was required, including treatment of psychiatric symptoms, support with legal and ethical issues, and provision of professional care. It is important to develop care models that are responsive to the complex needs of these rare conditions.

## Data Availability

Not applicable.
